# Lower Serum SCCA2 Detectability in Chronic Spontaneous Compared with Chronic Inducible Urticaria: A Prospective Observational Study

**DOI:** 10.3390/diagnostics16203367

**Published:** 2026-10-16

**Authors:** Osman Gökdeniz, Elif Çetinkaya, Burak Ünlü, Burak Yıldız, İbrahim Halil Yavuz, Nazlı Caf, Zafer Türkoğlu

**Affiliations:** 1 Department of Dermatology, Başakşehir Çam and Sakura City Hospital, 34480 İstanbul, Türkiye; ihalilyavuz@gmail.com (İ.H.Y.); cemzalu@gmail.com (Z.T.); 2 Department of Dermatology, Sultangazi Haseki Training and Research Hospital, 34265 İstanbul, Türkiye; 3 Department of Dermatology, Bakırköy Dr. Sadi Konuk Training and Research Hospital, 34147 İstanbul, Türkiye; elif.cetinkaya11@gmail.com; 4 Department of Dermatology, Bağcılar Training and Research Hospital, 34200 İstanbul, Türkiye; brkunlu95@gmail.com; 5 Department of Dermatology, Gaziosmanpaşa Training and Research Hospital, 34255 İstanbul, Türkiye; yildizburak004@gmail.com; 6 Department of Dermatology, Liv Hospital Ulus, 34340 İstanbul, Türkiye; drnazlicaf@gmail.com

**Keywords:** chronic inducible urticaria, chronic spontaneous urticaria, SCCA2, biomarkers

## Abstract

**Background/Objectives:** Squamous cell carcinoma antigen 2 (SCCA2; SERPINB4) is an IL-4/IL-13-inducible epithelial serpin that can inhibit human mast-cell chymase, providing a potential link to mast-cell-mediated inflammation. To our knowledge, SCCA2 has not previously been studied in chronic urticaria; we hypothesized that circulating SCCA2 would differ among chronic spontaneous urticaria (CSU), chronic inducible urticaria (CIndU), and healthy controls. **Methods:** This prospective, single-center observational study included 36 patients with CSU, 35 with provocation-confirmed CIndU, and 45 healthy controls; atopic or allergic diseases were excluded in all groups. Baseline blood sampling was performed before treatment initiation. Serum SCCA2 was measured by ELISA. Because quantification below the lower limit of quantification (LLOQ, 0.16 ng/mL) was not validated, primary interpretation used assay-defined categories based on the limit of detection (LOD, 0.09 ng/mL) and LLOQ; continuous analyses were exploratory. **Results:** SCCA2 detectability was 22.2% in CSU, 62.9% in CIndU, and 68.9% in controls. Detectability was lower in CSU than in CIndU and controls (Holm-adjusted *p* = 0.0015 and *p* < 0.001), whereas CIndU and controls did not differ (*p* = 0.637). Among patients, below-LOD SCCA2 was associated with CSU (OR 5.92, 95% CI 2.09–16.81) and remained associated after adjustment for total IgE, eosinophil count, and CRP (aOR 4.73, 95% CI 1.59–14.08; *p* = 0.005). **Conclusions:** Serum SCCA2 showed a CSU-associated low-detectability pattern, whereas CIndU and controls did not differ significantly. Because most estimates were below the LLOQ, findings represent differences in assay-defined detectability rather than validated concentrations. Independent validation is required.

## 1. Introduction

Chronic urticaria (CU) is characterized by recurrent wheals and/or angioedema persisting for >6 weeks. It is clinically classified into chronic inducible urticaria (CIndU), in which symptoms are triggered by specific, reproducible stimuli, and chronic spontaneous urticaria (CSU), which occurs without an identifiable external trigger. CU substantially impairs quality of life and imposes a meaningful socioeconomic burden [1,2].

Although the pathogenesis of CSU is not fully elucidated, two principal immunologic mechanisms have been described: IgE-mediated autoallergy (type I autoimmunity) and IgG-mediated type IIb autoimmunity [3]. In addition, accumulating evidence indicates that T-helper 2 (Th2)-mediated inflammation also contributes to CSU pathogenesis. IL-4 and IL-13 promote IgE class switching and may enhance mast-cell activation [4]. Findings for type 2-associated cytokines in peripheral-blood studies of CSU are heterogeneous, whereas lesional-skin studies more consistently demonstrate increased type 2-associated cytokine expression [5,6]. Circulating type 2-related biomarkers may show discordant patterns in CSU. In one cohort, serum periostin, an IL-4/IL-13-induced downstream molecule, was lower in patients with CSU than in healthy controls despite higher serum IL-13 levels, and periostin was not significantly correlated with IL-13 [7]. These findings indicate that circulating downstream biomarkers do not necessarily parallel circulating type 2 cytokine levels.

Although mast-cell activation is central to both CSU and CIndU, their underlying mechanisms remain incompletely characterized and need not be identical; CIndU itself comprises multiple stimulus-defined subtypes [1,8]. Squamous cell carcinoma antigen 2 (SCCA2; SERPINB4) is a clade B (ovalbumin) serpin and endogenous protease inhibitor [9]. SCCA2 can inhibit human mast-cell chymase and form SDS-stable complexes with the enzyme [10]. This interaction is potentially relevant to urticaria because in animal models chymase has been shown to potentiate histamine-induced wheal formation and induce a prolonged increase in microvascular permeability [11,12]; however, the in vivo significance of SCCA2-mediated chymase inhibition in human skin, particularly in chronic urticaria, remains unknown. SCCA2 expression in epithelial cells, including keratinocytes, is inducible by IL-4 and IL-13 [13]. Increased circulating and/or tissue SCCA2/SERPINB4 has been reported in atopic dermatitis, psoriasis, lichen planus, eosinophilic esophagitis, and squamous-cell carcinomas (Table 1) [14,15,16,17,18,19,20]. Whether circulating SCCA2 is altered in chronic urticaria, a predominantly mast-cell-driven disorder, remains unclear.

On this basis, we hypothesized that circulating SCCA2 would differ among CSU, CIndU, and healthy controls and examined its detectability and distribution across these groups. Healthy controls provided a reference context for interpreting differences between the patient groups. Continuous concentration-based and discriminatory analyses were performed as exploratory assessments.

## 2. Materials and Methods

### 2.1. Study Design and Participants

This prospective observational study was conducted at the Dermatology Outpatient Clinic of Başakşehir Çam and Sakura City Hospital, İstanbul, Türkiye, between 16 June and 15 October 2024.

Seventy-one patients with CU were included (CSU, *n* = 36; CIndU, *n* = 35), along with 45 healthy controls recruited to achieve broadly comparable age and sex distributions without individual matching. Although symptom duration varied, the diagnosis and formal classification as CSU or CIndU were established at study enrollment. Sex was recorded as a biological variable from the medical record and is reported as male/female; gender identity and race/ethnicity were not collected.

### 2.2. Classification of Chronic Urticaria Subtypes

Patients clinically considered to have CSU underwent FricTest^®^ (Moxie GmbH, Berlin, Germany) and TempTest^®^ (Courage + Khazaka electronic GmbH, Cologne, Germany) as limited classification checks intended to reduce potential misclassification by identifying otherwise unrecognized inducible features. Patients with no history suggestive of inducible triggers and negative results on both tests were classified as having CSU. Patients without a suggestive history who had a positive FricTest^®^ and/or TempTest^®^ result were excluded because overlapping spontaneous and inducible features were an exclusion criterion.

Patients with a history suggestive of CIndU underwent subtype-specific standardized provocation testing in accordance with current international urticaria guidelines, using FricTest^®^, TempTest^®^, and/or additional standardized challenges as appropriate [1,21,22]. Testing was performed using predefined exposure conditions and reading times. Second-generation H1-antihistamines were discontinued for at least 7 days before provocation testing, and a wheal response at the challenge site was considered a positive result. Only patients with positive subtype-specific provocation test results were classified as having CIndU. Accordingly, the analytical cohort comprised patients with CSU defined by the absence of a suggestive inducible-trigger history together with negative FricTest^®^ and TempTest^®^ results, and patients with provocation-confirmed CIndU. A schematic overview of the analytical cohort is provided in Figure 1.

### 2.3. Inclusion and Exclusion Criteria

Adults aged 18–65 years with recurrent wheals and/or angioedema persisting for >6 weeks were eligible. CSU and CIndU classification followed the approach described above. All participants provided written informed consent and were able to complete study questionnaires.

Patients were excluded if they had overlapping CSU and CIndU features; comorbid atopic or allergic diseases, including atopic dermatitis, asthma, or allergic rhinitis; psoriasis or other chronic inflammatory skin diseases; acute or chronic infection; systemic inflammatory disease or systemic autoimmune disease; immunodeficiency; malignancy; pregnancy or lactation; urticarial vasculitis; isolated angioedema; congenital/genetic disorders; or if they had received systemic immunosuppressive or immunomodulatory therapy within the preceding month. Autoimmune thyroid disease and asymptomatic autoantibody positivity were not exclusion criteria.

Healthy controls aged 18–65 years without a history of chronic urticaria; atopic dermatitis, asthma, allergic rhinitis, psoriasis, or other chronic inflammatory skin diseases; systemic inflammatory or autoimmune disease; immunodeficiency; or malignancy were recruited as a reference group. Individuals receiving systemic immunosuppressive or immunomodulatory therapy were not eligible as healthy controls.

### 2.4. Clinical Assessment

Disease duration was defined as the interval from patient-reported onset of recurrent urticaria symptoms to study enrollment. Disease control was assessed using the Urticaria Control Test (UCT); scores < 12 indicated uncontrolled disease and ≥12 controlled disease. Because treatment was initiated at enrollment, the UCT was administered one month later to reflect disease control during the preceding four weeks. Health-related quality of life was evaluated using the Dermatology Life Quality Index (DLQI) at diagnosis.

### 2.5. Blood Sample Collection and Routine Laboratory Measurements

Peripheral blood was collected after overnight fasting, at a similar time of day, before treatment initiation and, when applicable, before provocation testing. Sampling was performed in the absence of visible active wheals. Routine measurements included complete blood count with neutrophil, lymphocyte, eosinophil, and platelet counts, C-reactive protein (CRP), and total IgE; the neutrophil-to-lymphocyte ratio (NLR) and platelet-to-lymphocyte ratio (PLR) were calculated from absolute counts.

SCCA2 was measured in duplicate using a commercial enzyme-linked immunosorbent assay (ELISA) kit (Elabscience E-EL-H1722, Houston, TX, USA); absorbance was read at 450 nm, and instrument-derived SCCA2 estimates were calculated from the standard curve. The manufacturer-reported limit of detection (LOD) was 0.09 ng/mL, and the validated quantitative range was 0.16–10 ng/mL. Although manufacturer-provided sensitivity verification yielded an experimental LOD of 0.035 ng/mL, the manufacturer-reported LOD of 0.09 ng/mL was used as the classification threshold as a conservative analytical choice, thereby limiting interpretation of very low-level assay signals as detectable SCCA2. Values < 0.09 ng/mL were classified as below the LOD, 0.09–<0.16 ng/mL as detectable but below the lower limit of quantification (LLOQ), and ≥0.16 ng/mL as within the validated quantitative range. Values below the LLOQ were not interpreted as validated absolute concentrations. Continuous instrument-derived estimates, including values below the LLOQ, were retained only for exploratory rank-based, receiver operating characteristic (ROC), and sensitivity analyses.

All 116 samples were assayed in duplicate on three plates from a single kit lot. Samples were allocated to plates by block randomization stratified by study group, with comparable proportions of CSU, CIndU, and control samples on each plate. The intra-assay coefficient of variation (CV) was calculated from duplicate optical density readings overall and separately for samples with mean SCCA2 estimates between 0.06 and 0.12 ng/mL, encompassing the region surrounding the LOD. Replicate-level agreement in classification relative to the 0.09 ng/mL LOD was assessed. As assay-level sensitivity analyses, the CSU–CIndU detectability comparison was repeated using each replicate separately, and assay plate was additionally entered into the multivariable patient-only model.

### 2.6. Statistical Analysis

Statistical analyses were performed using IBM SPSS Statistics version 27 (IBM Corp., Armonk, NY, USA). Continuous variables were summarized as mean ± standard deviation or median (interquartile range), and categorical variables as *n* (%). An a priori G*Power 3.1.9 calculation indicated that 68 patients (34 per group) were required to detect a large effect (Cohen’s d = 0.8) with 90% power and a two-sided α of 0.05, based on the originally planned comparison of continuous SCCA2 estimates. Following written clarification from the manufacturer regarding the LOD–LLOQ distinction, assay-defined detectability categories guided the primary interpretation, whereas continuous-value analyses were exploratory. Primary inference did not rely on fixed-value substitution, which can bias estimates when below-LOD observations are frequent [23,24].

The overall distribution of the three assay-defined SCCA2 categories described in Section 2.5 was compared across CSU, CIndU, and controls using the Fisher–Freeman–Halton exact test. Pairwise comparisons of LOD-based detectability (≥0.09 vs. <0.09 ng/mL) used Fisher’s exact test with Holm adjustment. Wilson 95% confidence intervals (CIs) were calculated for group-specific proportions and Newcombe intervals for absolute differences.

In the patient-only comparison, the association between below-LOD SCCA2 and CSU was summarized using an odds ratio (OR) with a 95% CI. A secondary multivariable logistic regression used CSU versus CIndU as the dependent variable and below-LOD SCCA2, total IgE, eosinophil count, and CRP as simultaneous predictors. Total IgE and CRP were natural log-transformed because of right-skewed distributions; adjusted ORs (aORs) were expressed per two-fold increase for these variables and per 0.1 × 10^3^/µL increase for eosinophil count. Because smoking status differed across groups, a separate patient-only sensitivity analysis adjusted the below-LOD SCCA2–CSU association for age, sex, and ever-smoking status (current or former vs. never; Supplementary Table S2).

Exploratory comparisons of instrument-derived SCCA2 estimates used the Kruskal–Wallis test followed by Dunn’s test with Holm adjustment. Age was approximately normally distributed and was compared across groups using one-way ANOVA; other continuous variables were compared between CSU and CIndU using the Mann–Whitney U test, and categorical variables using the chi-square or Fisher’s exact test, as appropriate. Exploratory ROC analysis assessed discrimination between CSU and CIndU using continuous instrument-derived SCCA2 estimates, with CSU as the positive state and lower estimates indicating CSU. The 95% confidence interval for the area under the curve (AUC) was estimated by bootstrapping (2000 resamples). Sensitivity analyses replaced below-LOD estimates with LOD/2, LOD/√2, or 0. No clinical decision threshold was inferred.

Within each patient group, Spearman’s rank correlation assessed associations of SCCA2 with total IgE, eosinophil count, CRP, UCT, and DLQI. Baseline SCCA2 estimates were compared between controlled and uncontrolled disease at one month using the Mann–Whitney U test. These exploratory analyses were not adjusted for multiple testing. A complementary patient-only sensitivity analysis modeled ln-transformed SCCA2 with ln-total IgE, eosinophil count, and ln-CRP to assess only the directional consistency of the CSU–CIndU association (Supplementary Table S1).

### 2.7. Ethical Considerations

The study was approved by the Clinical Research Ethics Committee of Başakşehir Çam and Sakura City Hospital (approval no. KAEK/29.05.2024.16) and conducted in accordance with the Declaration of Helsinki. All participants provided written informed consent before enrollment.

## 3. Results

### 3.1. Participant Characteristics

A total of 71 patients with CU (CSU, *n* = 36; CIndU, *n* = 35) and 45 healthy controls were included (Figure 1). Table 2 summarizes baseline characteristics; age and sex did not differ across groups (all *p* > 0.05), whereas smoking status did.

Table 3 summarizes clinical and laboratory characteristics. Disease duration, UCT, DLQI, total IgE, eosinophil count, CRP, NLR, and PLR did not differ between CSU and CIndU (all *p* > 0.05). Angioedema occurred in 17/36 patients with CSU (47.2%) and none with CIndU (*p* < 0.001).

### 3.2. Serum SCCA2 Detectability and Distribution Across CSU, CIndU, and Controls

The overall distribution of the three assay-defined SCCA2 categories differed significantly among CSU, CIndU, and controls (Fisher–Freeman–Halton *p* < 0.001) (Table 4). LOD-based detectability (≥0.09 ng/mL) was 22.2% (95% CI, 11.7–38.1%) in CSU, 62.9% (95% CI, 46.3–76.8%) in CIndU, and 68.9% (95% CI, 54.3–80.5%) in controls. Detectability was lower in CSU than in CIndU (Holm-adjusted *p* = 0.0015) and controls (Holm-adjusted *p* < 0.001), whereas CIndU and controls did not differ (Holm-adjusted *p* = 0.637).

Exploratory rank-based analysis of instrument-derived SCCA2 estimates also showed differences among groups (Kruskal–Wallis *p* < 0.001) (Table 4 and Figure 2). Dunn’s post-hoc test with Holm adjustment showed lower estimates in CSU than in CIndU and controls (both *p* < 0.001), with no significant difference between CIndU and controls. Because most estimates were below the LLOQ, these findings were interpreted as rank-based distributional differences rather than precise concentration differences.

Replicate-level classification relative to the 0.09 ng/mL LOD was concordant in 110 of 116 samples (94.8%). The overall intra-assay CV was 6.2% and was 11.4% among samples with mean SCCA2 estimates between 0.06 and 0.12 ng/mL. Repeating the CSU–CIndU detectability analysis using each replicate separately yielded similar group differences, which remained statistically significant in both replicate-specific analyses. Additional adjustment for assay plate did not materially alter the association between below-LOD SCCA2 and CSU (aOR, 4.60; 95% CI, 1.50–13.90).

### 3.3. Low SCCA2 Detectability in CSU Compared with CIndU

In the patient-only comparison, below-LOD SCCA2 was 40.6 percentage points more frequent in CSU than in CIndU (95% CI, 17.7–58.1), corresponding to an unadjusted OR of 5.92 (95% CI, 2.09–16.81) for CSU among patients with below-LOD SCCA2.

In the secondary multivariable analysis, below-LOD SCCA2 remained associated with CSU after adjustment for total IgE, eosinophil count, and CRP (aOR, 4.73; 95% CI, 1.59–14.08; *p* = 0.005) (Table 5). The association was also maintained in the separate sensitivity analysis adjusting for age, sex, and ever-smoking status (aOR, 7.17; 95% CI, 2.25–22.87; *p* < 0.001; Supplementary Table S2).

Exploratory ROC analysis, with CSU defined as the positive state and lower SCCA2 estimates indicating CSU, yielded an AUC of 0.77 (bootstrap 95% CI, 0.66–0.88; *p* < 0.001). Replacing below-LOD values with LOD/2, LOD/√2, or 0 yielded an AUC of 0.74 with each approach, indicating that discrimination was not entirely dependent on the ordering of below-LOD estimates.

### 3.4. Association of SCCA2 with Quality of Life, Short-Term Disease Control, and Conventional Laboratory Parameters

Within both patient groups, baseline instrument-derived SCCA2 estimates were not significantly associated with DLQI at diagnosis or UCT at one month; across the patient cohort, estimates did not differ between controlled and uncontrolled disease at one month (all *p* > 0.05). SCCA2 estimates were not significantly correlated with total IgE or CRP in either patient group (all *p* > 0.05). An inverse correlation with eosinophil count reached nominal significance in CIndU (Spearman’s r = −0.355, *p* = 0.036) but not in CSU (r = −0.294, *p* = 0.082); given the number of exploratory comparisons and the absence of multiplicity adjustment, this finding should be interpreted cautiously. Results of the complementary exploratory continuous-value regression are provided in Supplementary Table S1.

## 4. Discussion

In this prospective study, the principal finding was markedly reduced serum SCCA2 detectability in CSU. Detectability was significantly lower in CSU than in both CIndU and healthy controls, whereas CIndU and controls did not differ. In the patient-only analysis, below-LOD SCCA2 remained associated with CSU after adjustment for total IgE, eosinophil count, and CRP. The absence of a significant difference between CIndU and healthy controls suggests that the observed group difference is primarily characterized by reduced circulating SCCA2 detectability in CSU rather than increased SCCA2 as a feature of CIndU.

Because most SCCA2 estimates were below the LLOQ and many were close to the LOD, the principal finding should be interpreted as a difference in assay-defined detectability rather than as a validated quantitative concentration difference. Some classification uncertainty around the LOD therefore remains. However, replicate-level classification was highly concordant, and the CSU–CIndU finding was maintained in replicate-specific analyses and after adjustment for assay plate. Exploratory rank-based and ROC analyses were directionally consistent but were considered supportive rather than threshold-defining, and no clinical cut-off was inferred. Confirmation with a higher-sensitivity assay is required.

The biological basis of reduced circulating SCCA2 detectability in CSU remains uncertain. One potentially relevant context is the protease-inhibitory function of SCCA2. SCCA2 can inhibit human mast-cell chymase and form SDS-stable complexes with the enzyme, whereas in animal models chymase has been shown to potentiate histamine-induced wheal formation and increase microvascular permeability [10,11,12]. These observations provide a biological rationale for considering SCCA2 in a mast-cell-driven disorder such as urticaria, although chymase-related mechanisms were not evaluated here.

A second relevant context is the regulation of SCCA2 by type 2 cytokines. As outlined above, circulating type 2-associated markers in CSU may diverge from local cutaneous inflammatory activity [5,6,7]. Because IL-4 and IL-13 can induce SCCA2 expression in keratinocytes [13], cutaneous SCCA2 expression might be increased in lesional urticarial skin; this has not been examined. In the skin diseases in which both tissue expression and serum concentrations have been studied, the two have been reported to change in the same direction [14,15]. The present data do not establish a dissociation between cutaneous and circulating SCCA2 in urticaria; determining whether one occurs will require paired tissue and serum measurements.

The presence of measurable serum SCCA2 in healthy controls is biologically plausible. Although SCCA2 is primarily intracellular and lacks a classical signal sequence, endoplasmic reticulum/Golgi-independent release has been reported [9]. In normal adult tissues, SCCA1 and SCCA2 are constitutively expressed in several non-cutaneous stratified squamous epithelia, whereas interfollicular epidermis shows little or no immunoreactivity in normal skin and becomes strongly positive mainly where it is inflamed or thickened [20]. Thus, detectable serum SCCA2 in healthy individuals need not imply active cutaneous inflammation; constitutive expression in non-cutaneous squamous epithelia provides one potential source of the basal circulating pool, although its tissue sources remain undefined. Consistent with this, measurable but low serum SCCA2 has been reported in healthy control groups, with medians ranging from 0.14 ng/mL in 25 healthy volunteers (range, 0.07–0.27; 68% below 0.15 ng/mL) [19] to approximately 0.7 ng/mL in the healthy controls of the atopic dermatitis and psoriasis studies summarized in Table 1 [14,15]. In the present control group, 30 of 45 instrument-derived estimates (66.7%) fell below the LLOQ of 0.16 ng/mL (Table 4), closely comparable to the proportion reported in healthy volunteers by Yasumatsu et al. [19].

The epithelial origin of SCCA2 also provides context for the comparison with other inflammatory skin diseases. Elevated circulating SCCA2 and increased lesional SCCA2 expression have been reported in atopic dermatitis and psoriasis (Table 1) [14,15]. Both conditions are characterized by prominent epidermal involvement. By contrast, urticaria is predominantly characterized by upper-dermal edema and perivascular–interstitial inflammation, with comparatively limited epidermal involvement [25]. This difference may help explain why the marked serum elevations reported in those conditions were not observed in either urticaria group.

This explanation, however, applies equally to CSU, in which epidermal involvement is similarly limited. It is therefore consistent with the absence of an elevation in both urticaria groups, but it does not by itself account for the further reduction observed in CSU relative to both CIndU and healthy controls. SCCA2 biology is not confined to its epithelial origin, since it is also an endogenous inhibitor of mast-cell chymase and is regulated by type 2 cytokines [10,13]; the CSU-associated reduction therefore suggests that additional factors, which would have to differ between CSU and CIndU, influence circulating SCCA2 detectability. Their nature remains unresolved.

Taken together, chymase inhibition, cytokine-regulated expression, and the epithelial origin of SCCA2 provide plausible biological contexts for the observed circulating pattern, but none was directly tested. SCCA2 is primarily an intracellular epithelial protein rather than a conventionally secreted soluble mediator distributed between tissue and blood compartments [9,20]. For an intracellular protein such as SCCA2, increased tissue expression cannot be assumed to be reflected in circulating concentrations, because appearance in serum additionally requires release from the intracellular pool [9]. Chymase activity, SCCA2–chymase complexes, and tissue SCCA2 expression were not measured in the present study. No specific mechanism for the low circulating detectability observed in CSU can therefore be proposed.

Direct comparisons of CSU and CIndU have reported heterogeneous findings for conventional laboratory markers: Kumaran et al. found no significant difference in total IgE, although CRP was higher in CSU, whereas Chen et al. likewise reported no significant difference in total IgE [26,27]. A recent study of circulating immunometabolic and barrier-associated markers in chronic urticaria also found no differences between spontaneous and inducible forms, although the inducible subgroup was small (*n* = 5) [28]. In our cohort, in which chronic urticaria was diagnosed and classified at enrollment and baseline sampling preceded treatment initiation, total IgE, eosinophil count, and CRP did not differ between CSU and CIndU, and adjustment for these variables did not account for the association between below-LOD SCCA2 and CSU. Blood-count-derived inflammatory indices have also been examined in CSU. In a recent cohort of 246 patients, NLR was not associated with UAS7 or with response to standard-dose antihistamines [29], and in two omalizumab studies, NLR and PLR did not distinguish treatment-response groups or appear useful for predicting treatment efficacy [30,31]. Neither NLR nor PLR differed between CSU and CIndU (Table 3). Whether SCCA2 detectability provides clinically useful information beyond existing assessment cannot be determined from the present study and requires prospective validation in diagnostically uncertain patients, among whom provocation testing was positive in fewer than 60% of referred patients in one tertiary cohort [32].

Serum SCCA2 was not associated with DLQI at diagnosis or UCT-based disease control one month after treatment initiation, providing no evidence for a straightforward relationship with quality-of-life impairment or short-term disease control. Because UCT was assessed after treatment initiation, however, the present study cannot determine whether SCCA2 varies with untreated baseline disease activity.

The present findings may also inform the interpretation of circulating type 2-associated biomarkers in CSU. A similar unexpected pattern has now been observed for two IL-4/IL-13-inducible downstream molecules in CSU: reduced serum periostin, which was not significantly correlated with IL-13 [7], and markedly reduced SCCA2 detectability in the present study. Set against evidence of increased type 2-associated activity in lesional CSU skin [5], these observations suggest that circulating downstream biomarkers may not directly parallel local cutaneous type 2-associated activity. Future biomarker studies in CSU should therefore avoid assuming that the circulating abundance of a type 2-associated downstream molecule is a direct surrogate of cutaneous pathway activity.

This study has several limitations. The single-center design may limit generalizability, and the modest sample size precluded adequately powered analyses of individual CIndU subtypes. Systematic provocation testing for all CIndU subtypes was not performed in patients clinically considered to have CSU; therefore, unrecognized CIndU cannot be completely excluded. The numbers excluded during screening because of either a positive FricTest^®^/TempTest^®^ result without a suggestive history or a negative subtype-specific provocation test despite a suggestive history were not systematically recorded. Because patients with concomitant spontaneous and inducible features were excluded, the generalizability of these findings to patients with overlapping CSU and CIndU is limited. Limited quantification below the LLOQ and uncertainty near the detection threshold remain important analytical limitations. SCCA2 was assessed at a single time point, precluding evaluation of longitudinal changes or treatment-related dynamics; baseline UAS7 in CSU, tissue SCCA2 expression, chymase activity, SCCA2–chymase complexes, and CSU immunologic endotypes, including autoantibody status, were not evaluated. Larger multicenter studies with repeated measurements, standardized clinical classification, and paired serum–tissue analyses are needed to validate the observed SCCA2 detectability pattern and determine its biological and clinical relevance.

## 5. Conclusions

The principal insight from this study is that circulating SCCA2 does not conform to a simple model in which serum SCCA2 directly reflects epidermal or type 2-associated cutaneous inflammatory activity. The similarity between CIndU and healthy controls indicates that the distinctive finding was selectively reduced detectability in CSU rather than a generalized alteration across chronic urticaria. This pattern cannot be explained solely by the limited epidermal involvement shared by CSU and CIndU and was not accounted for by total IgE, eosinophil count, or CRP. Together with the presence of measurable circulating SCCA2 in healthy individuals and the constitutive expression of SCCA2 in normal non-cutaneous epithelia, these findings indicate that circulating SCCA2 is not specific to inflamed skin and should be interpreted in the context of its broader epithelial biology. Given that most estimates were below the assay LLOQ and tissue SCCA2 was not measured, these findings should be considered hypothesis-generating and require confirmation with assays of greater low-end sensitivity.

## Figures and Tables

**Figure 1:**
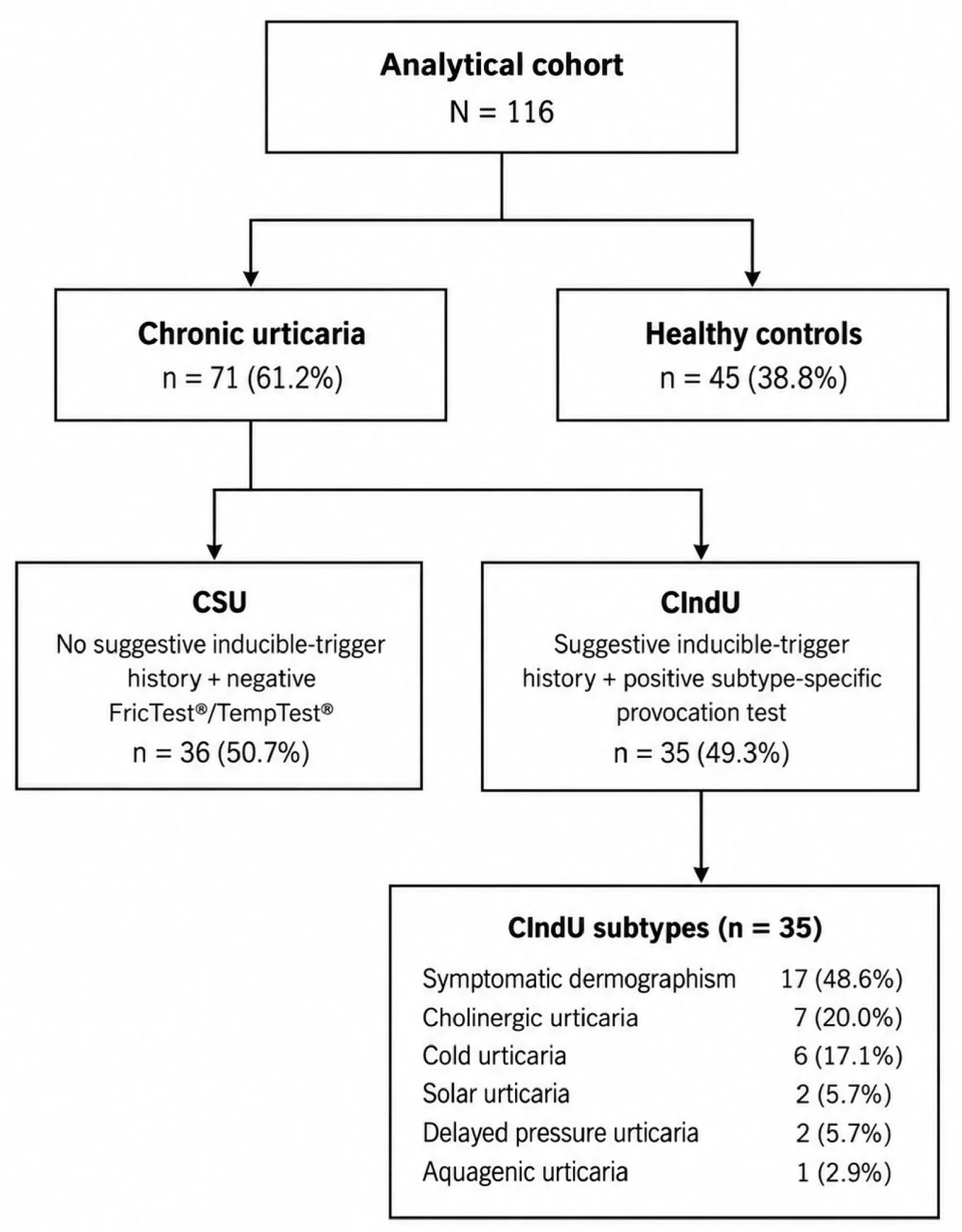
Analytical cohort and clinical classification. Percentages for chronic urticaria and healthy controls are based on the analytical cohort (*N* = 116); those for CSU and CIndU on patients with chronic urticaria (*n* = 71); and those for CIndU subtypes on patients with CIndU (*n* = 35). CSU, chronic spontaneous urticaria; CIndU, chronic inducible urticaria.

**Figure 2:**
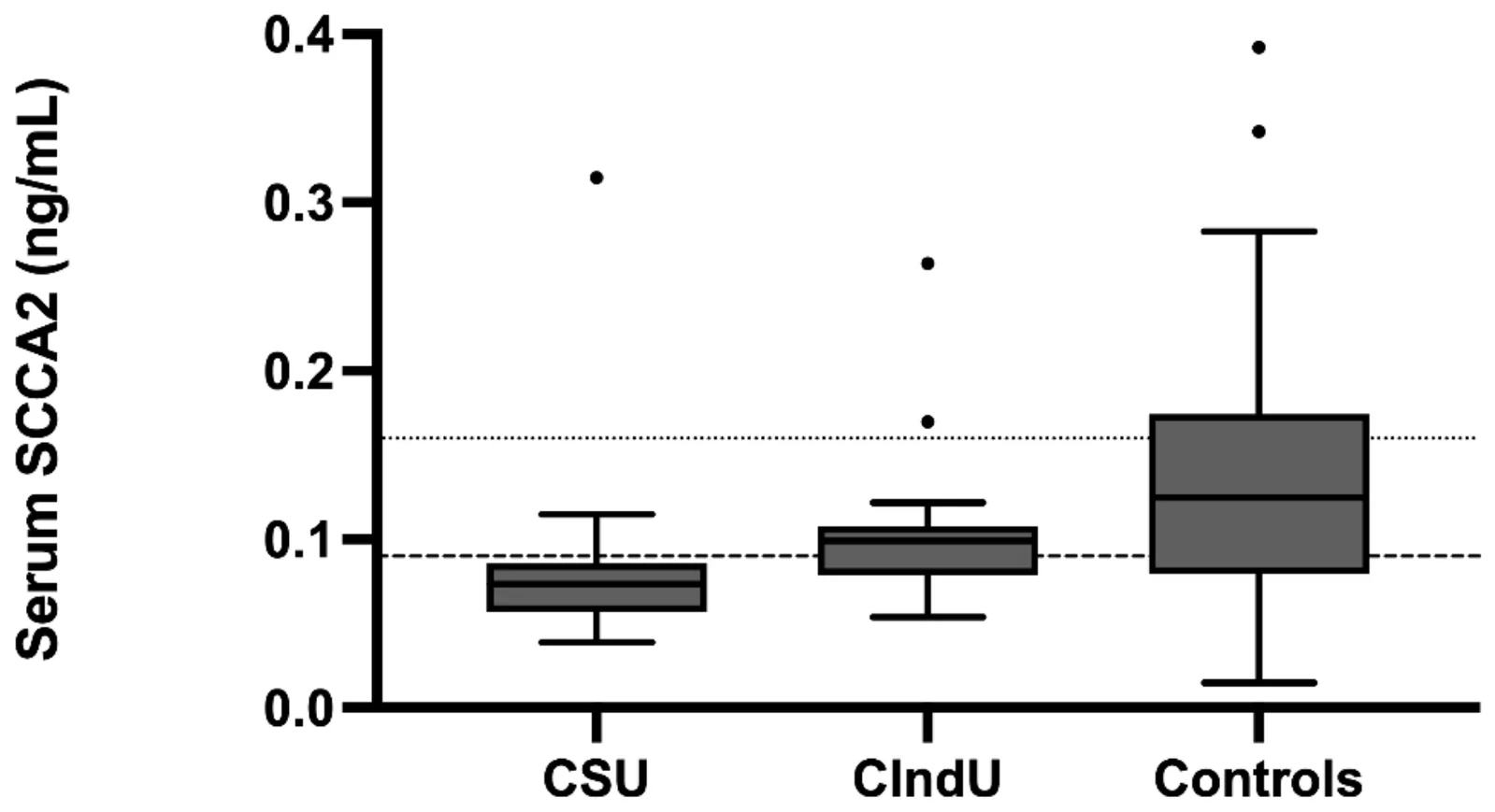
Distribution of instrument-derived serum squamous cell carcinoma antigen 2 (SCCA2) estimates among patients with chronic spontaneous urticaria (CSU), chronic inducible urticaria (CIndU), and healthy controls. Box plots depict the median and interquartile range (IQR), with whiskers extending to 1.5 × IQR; individual points represent outliers. The dashed horizontal line indicates the manufacturer-reported limit of detection (LOD, 0.09 ng/mL), and the dotted horizontal line indicates the lower limit of quantification (LLOQ, 0.16 ng/mL).

**Table 1:** Representative reports of circulating and tissue SCCA2/SERPINB4 across selected diseases.

Condition	Circulating SCCA2/SERPINB4	Tissue/Expression Findings	Representative Reference(s)
**Atopic dermatitis**	Serum SCCA2 increased compared with healthy/non-allergic controls and associated with disease severity and treatment response	SCCA2 was strongly expressed in the lesional epidermis, particularly from the spinous to granular layers	Okawa et al., 2018 [14]
**Psoriasis**	Serum SCCA2 was increased compared with healthy controls, correlated with disease severity, and decreased with clinical improvement	Increased SCCA2 staining was observed in lesional but not nonlesional skin	Watanabe et al., 2016 [15]
**Lichen planus**	Serum SCCA2 was increased in patients with lichen planus compared with controls	Not assessed in that study	Khattab & Samir, 2020 [16]
**Eosinophilic esophagitis**	Serum SCCA2 was increased compared with controls, although substantial overlap was present	SERPINB4 upregulated in affected esophageal tissue	Rochman et al., 2017 [17]; Ishihara et al., 2017 [18]
**Squamous-cell carcinoma**	Serum SCCA2 increased in head-and-neck SCC and associated with disease progression	SCCA2/SERPINB4 expression has been documented in squamous-cell carcinoma tissue	Yasumatsu et al., 2019 [19]; Cataltepe et al., 2000 [20]

SCCA2, squamous cell carcinoma antigen 2; SERPINB4, serpin family B member 4; SCC, squamous-cell carcinoma. SCCA2 corresponds to SERPINB4; terminology is reported according to the individual studies. Absolute circulating concentrations should not be directly compared across studies because analytical platforms, assay calibration, study populations, and the measured SCCA species differ.

**Table 2:** Demographic characteristics of patients with chronic urticaria and healthy controls.

Variable		CSU (*n* = 36)	CIndU (*n* = 35)	Healthy Controls (*n* = 45)	*p* Value
		*n* (%)	*n* (%)	*n* (%)	
**Age,** **years, mean ± SD**		33.97 ± 9.89	35.51 ± 10.93	33.11 ± 9.44	0.568
**Sex,** ***n*** **(%)**					0.683
	Male	12 (33.3)	15 (42.9)	16 (35.6)	
	Female	24 (66.7)	20 (57.1)	29 (64.4)	
**Smoking status,** ***n*** **(%)**					0.016
	Current smoker	13 (36.1)	15 (42.9)	10 (22.2)	
	Former smoker	1 (2.8)	4 (11.4)	0	
	Never smoker	22 (61.1)	16 (45.7)	35 (77.8)	
**Alcohol consumption,** ***n*** **(%)**					0.291
	Social drinking	6 (16.7)	11 (31.4)	9 (20)	
	No alcohol use	30 (83.3)	24 (68.6)	36 (80)	

**Abbreviations:** ANOVA, analysis of variance; CSU, chronic spontaneous urticaria; CIndU, chronic inducible urticaria; SD, standard deviation. Data are presented as mean ± SD or *n* (%). *p* values were calculated using one-way ANOVA for age. Categorical variables were compared using the chi-square test; Fisher’s exact test was used when expected cell counts were small.

**Table 3:** Clinical and routine laboratory characteristics of patients with CSU and CIndU.

Variable	CSU (*n* = 36)	CIndU (*n* = 35)	*p* Value
Disease duration, months, median (IQR)	12.0 (5.5–39.0)	36.0 (10.5–90.0)	0.068
Angioedema, *n*/*N* (%)	17/36 (47.2)	0/35 (0.0)	<0.001
UCT score at 1-month follow-up, median (IQR)	6.0 (3.0–8.8)	6.5 (4.0–11.8)	0.310
DLQI score at diagnosis, median (IQR)	9.0 (4.5–14.5)	10.0 (4.0–16.0)	0.711
Eosinophil count, ×10^3^/µL, median (IQR)	0.16 (0.12–0.26)	0.13 (0.10–0.18)	0.201
NLR, median (IQR)	1.82 (1.30–2.42)	1.60 (1.36–2.21)	0.483
PLR, median (IQR)	117.2 (102.7–131.5)	118.1 (90.0–130.4)	0.654
CRP, mg/L, median (IQR)	2.75 (1.20–6.00)	1.80 (0.80–4.05)	0.180
Total IgE, IU/mL, median (IQR)	118.0 (45.3–224.5)	152.0 (90.7–281.0)	0.182

Abbreviations: CIndU, chronic inducible urticaria; CRP, C-reactive protein; CSU, chronic spontaneous urticaria; DLQI, Dermatology Life Quality Index; IgE, immunoglobulin E; IQR, interquartile range; UCT, Urticaria Control Test; NLR, neutrophil-to-lymphocyte ratio; PLR, platelet-to-lymphocyte ratio. Data are presented as median (interquartile range) or *n*/*N* (%). Continuous variables were compared using the Mann–Whitney U test, and angioedema frequency was compared using Fisher’s exact test.

**Table 4:** Serum SCCA2 estimates and assay-defined detectability categories in patients with CSU, CIndU, and controls.

Variable	CSU (*n* = 36)	CIndU (*n* = 35)	Controls (*n* = 45)	*p* Value
Serum SCCA2, median (IQR), ng/mL	0.074 (0.057–0.086)	0.099 (0.081–0.106)	0.125 (0.082–0.168)	<0.001
Below LOD, <0.09 ng/mL, *n* (%)	28 (77.8)	13 (37.1)	14 (31.1)	<0.001 *
Detectable but below LLOQ, 0.09–<0.16 ng/mL, *n* (%)	7 (19.4)	18 (51.4)	16 (35.6)	
Within validated quantitative range, 0.16–10 ng/mL, *n* (%)	1 (2.8)	4 (11.4)	15 (33.3)	

Abbreviations: CSU, chronic spontaneous urticaria; CIndU, chronic inducible urticaria; IQR, interquartile range; LOD, limit of detection; LLOQ, lower limit of quantification; SCCA2, squamous cell carcinoma antigen 2. Data are presented as median (IQR) or *n* (%). The manufacturer-reported LOD was 0.09 ng/mL, and the validated quantitative range of the assay was 0.16–10 ng/mL. Values from 0.09 to <0.16 ng/mL were classified as detectable but below the LLOQ. The *p* value for continuous instrument-derived SCCA2 estimates was calculated using the Kruskal–Wallis test. * The *p* value refers to the overall comparison of the three assay-defined SCCA2 categories across groups using the Fisher–Freeman–Halton exact test. Pairwise comparisons of LOD-based detectability were performed using Fisher’s exact test with Holm adjustment and are reported in the text. Percentages may not sum to 100% due to rounding.

**Table 5:** Association of below-LOD SCCA2 with CSU after adjustment for conventional laboratory parameters.

Variable	Adjusted OR (95% CI)	*p* Value
Below -LOD SCCA2, <0.09 vs. ≥0.09 ng/mL	4.73 (1.59–14.08)	0.005
Total IgE, per doubling	0.86 (0.64–1.16)	0.321
Eosinophil count, per 0.1 ×10^3^/µL increase	1.16 (0.70–1.93)	0.558
CRP, per doubling	1.14 (0.81–1.61)	0.463

The dependent variable was CSU versus CIndU; therefore, an OR > 1 indicates higher odds of CSU. Total IgE and CRP were natural log-transformed for modelling, with ORs expressed per doubling for ease of interpretation. All variables were entered simultaneously. The analysis included all 71 patients with chronic urticaria. Abbreviations: CI, confidence interval; CIndU, chronic inducible urticaria; CRP, C-reactive protein; CSU, chronic spontaneous urticaria; IgE, immunoglobulin E; LOD, limit of detection; OR, odds ratio; SCCA2, squamous cell carcinoma antigen 2.

## Data Availability

The de-identified dataset supporting the findings of this study is available from the corresponding author upon reasonable request.

## Supplementary Materials

The following supporting information can be downloaded at https://www.mdpi.com/article/10.3390/diagnostics16203367/s1: Table S1: Exploratory continuous-value sensitivity analysis; Table S2: Association of below-LOD SCCA2 with CSU after adjustment for age, sex, and ever-smoking status.
