# Supplementary material for: Lower Serum SCCA2 Detectability in Chronic Spontaneous Compared with Chronic Inducible Urticaria: A Prospective Observational Study
Source: Diagnostics (Basel). 2026 Oct 16;16(20):3367. doi: 10.3390/diagnostics16203367 (PMC13702944; doi:10.3390/diagnostics16203367)
Supplement: Supplementary file 1 [file diagnostics-16-03367-s001.zip › diagnostics-4609059-supplementary.pdf]

Supplementary Material

Exploratory continuous-value regression

Among patients with chronic urticaria, univariable and multivariable logistic regression analyses were performed using CSU versus CIndU as the dependent variable. Instrument-derived serum SCCA2 estimates, total IgE, eosinophil count, and CRP were included as independent variables. Because of right-skewed distributions, SCCA2, total IgE, and CRP were natural log-transformed, whereas eosinophil count was entered untransformed. Odds ratios with 95% confidence intervals were reported. No LOD-based substitution was applied in these regression models.

Supplementary Table S1. Exploratory continuous-value sensitivity analysis

Variable	Univariable OR (95% CI)	P value	Multivariable OR (95% CI)	P value
**ln-SCCA2**	**0.08 (0.01–0.47)**	**.006**	**0.10 (0.01–0.70)**	**.020**
**ln-total IgE**	**0.77 (0.53–1.12)**	**.174**	**0.81 (0.53–1.25)**	**.336**
**Eosinophil count, per 0.1 ×10^3/μL**	**1.37 (0.87–2.17)**	**.171**	**1.06 (0.65–1.75)**	**.800**
**ln-CRP**	**1.35 (0.87–2.13)**	**.175**	**1.18 (0.72–1.92)**	**.518**

The dependent variable was CSU versus CIndU; therefore, an OR >1 indicates higher odds of CSU. SCCA2, total IgE, and CRP were natural log-transformed. For ln-transformed variables, ORs represent a one-unit increase on the natural-log scale; for ln-SCCA2, this corresponds to an approximately 2.72-fold increase in the instrument-derived SCCA2 estimate. The OR for eosinophil count is reported per 0.1 ×10^3/μL increase. Below-LLOQ instrument-derived estimates were entered without substitution. Because these estimates lie outside the validated quantitative range, the ORs should not be interpreted as concentration–response effects; this analysis was used only to assess directional consistency when SCCA2 was modeled continuously without dichotomization at the 0.09 ng/mL LOD.

Abbreviations: CI, confidence interval; CIndU, chronic inducible urticaria; CRP, C-reactive protein; CSU, chronic spontaneous urticaria; IgE, immunoglobulin E; ln, natural logarithm; LOD, limit of detection; LLOQ, lower limit of quantification; OR, odds ratio; SCCA2, squamous cell carcinoma antigen 2.

Age-, sex-, and smoking-adjusted analysis of below-LOD SCCA2

Among patients with chronic urticaria, multivariable logistic regression was used to evaluate whether the association between below-LOD SCCA2 and CSU persisted after adjustment for age, sex, and ever-smoking status. CSU versus CIndU was used as the dependent variable. Below-LOD SCCA2 (<0.09 vs ≥0.09 ng/mL), age, sex, and smoking status were entered simultaneously as independent variables. Sex was coded as male versus female, and smoking status was dichotomized as ever-smoker (current or former) versus never-smoker. Adjusted odds ratios with 95% confidence intervals were reported. This exploratory analysis was intended to assess potential confounding by age, sex, and smoking status rather than diagnostic or predictive performance.

Supplementary Table S2. Association of below-LOD SCCA2 with CSU after adjustment for age, sex, and ever-smoking status

**Variable**	**Adjusted OR (95% CI)**	**P value**
Below-LOD SCCA2, <0.09 vs ≥0.09 ng/mL	7.17 (2.25–22.87)	<.001
Age, per 1-year increase	1.01 (0.95–1.06)	.758
Male sex vs female	1.11 (0.36–3.45)	.852
Ever-smoker vs never-smoker	0.40 (0.13–1.19)	.101

The dependent variable was CSU versus CIndU; therefore, an OR >1 indicates higher odds of CSU. All variables were entered simultaneously. Ever-smoking included current and former smoking. The analysis included all 71 patients with chronic urticaria.

Abbreviations: CI, confidence interval; CIndU, chronic inducible urticaria; CSU, chronic spontaneous urticaria; LOD, limit of detection; OR, odds ratio; SCCA2, squamous cell carcinoma antigen 2.
